# 183 Association between mental health and different patterns of physical activity in adolescents in Luxembourg – HBSC 2022

**DOI:** 10.1093/eurpub/ckae114.147

**Published:** 2024-09-26

**Authors:** Felipe Mendes, Joana Lopes Ferreira, Carolina Catunda

**Affiliations:** University of Luxembourg, Luxembourg, Luxembourg; University of Luxembourg, Luxembourg, Luxembourg; University of Luxembourg, Luxembourg, Luxembourg

## Abstract

**Purpose:**

Regular physical activity (PA) is a key protective factor for health in youths. The WHO recommends that adolescents do at least an average of 60 minutes per day of moderate-to-vigorous physical activity (MVPA) and incorporate vigorous-intensity aerobic activities (VPA) at least 3 days a week. Nonetheless, lower amounts of PA already carry benefits. This study aims to explore and to examine association between well-being and PA levels in adolescents in Luxembourg.

**Methods:**

A representative sample of 8117 adolescents (11-to-18-years-old) took part in the 2022 Health Behaviour in School-aged Children (HBSC) Luxembourg survey. Based on the WHO-5 Index adolescents were categorised as “depression symptomatology”, “low mood” and normal well-being. In addition, they were categorised as frequency per week (0 to 7 days) of MVPA and VPA practice. Multinomial logistic regressions were performed, controlling by sex and SES. Normal well-being and physical inactivity were the respective refence groups.

**Results:**

The results showed an association between mental well-being and both MVPA and VPA (p<.001). Compared to their inactive peers and independently of sex and SES, adolescents in Luxembourg who practiced MVPA 4 days per week or VPA 2 times per week had decreased odds to present low mood (ORMVPA=0.63, 95%CI[0.45-0.87] and ORVPA=0.75, 95%CI[0.60-0.92]) and PA even in a small frequency (1 day per week) already decreased the odds to have depressive symptoms (ORMVPA=0.67, 95%CI[0.51-0.89] and ORVPA=0.61, 95%CI[0.49-0.75]).

**Conclusions:**

These findings highlight the significance of physical activity in fostering well-being, revealing an inverse relationship between the frequency of physical activity and the occurrence of depressive symptomatology and low well-being. In this regard, the advantages of engaging in physical activity are linked not only to physiological benefits but also to social interactions within these practices. In this context, the benefits of regular physical activity extend beyond its direct impact on mental health, encompassing both physiological and social dimensions.

